# Adjunctive Use of Electrical Stimulation (E-Stim) for Pain Relief and Rehabilitation in a Skilled Nursing Facility Patient Following Right Glenoid Fracture and Humeral Head Dislocation

**DOI:** 10.7759/cureus.86690

**Published:** 2025-06-24

**Authors:** Dailyn A Rodriguez Reyes, Erin Kominami, Jeanney Kang, Navid Darouian

**Affiliations:** 1 Obstetrics and Gynecology, University of California Los Angeles David Geffen School of Medicine, Los Angeles, USA; 2 Rehabilitation Medicine, Fireside Health Care Center, Los Angeles, USA; 3 Internal Medicine, University of California Los Angeles David Geffen School of Medicine, Los Angeles, USA

**Keywords:** glenoid fracture, pain relief, pain relief options, skilled nursing facility care, transcutaneous electrical nerve stimulation

## Abstract

We describe the treatment approach of an 87-year-old woman who developed a right glenoid fracture with right humeral head dislocation during hospitalization before transferring to a skilled nursing facility (SNF). The focus lies on how electrical stimulation (e-stim) therapy serves as an essential component of rehabilitation protocols. E-stim proved effective for pain management and enhanced mobility while leading to better functional outcomes. The patient received therapy without complications and reached pain management milestones and showed steady advancement toward therapy targets. E-stim therapy demonstrates its value as a supportive treatment in the rehabilitation process of subacute orthopedic care, especially for older patients living in SNF settings.

## Introduction

Elderly patients with complex glenoid fractures and associated humeral head dislocations often face chronic pain, functional decline, and prolonged recovery periods. Effective management of these injuries requires coordinated care between acute hospital settings and post-acute care facilities. Treatment strategies for older adults must address not only anatomical healing but also the complications of immobility and the restoration of independence.

Electrical stimulation therapy (e-stim), which delivers low-voltage electrical currents to stimulate muscle contractions, has been shown to reduce pain, enhance local blood circulation, and support functional recovery. Widely used in rehabilitation - including stroke recovery and post-surgical orthopedic care - e-stim has demonstrated benefits in accelerating recovery and restoring musculoskeletal function [1,2]. In skilled nursing facilities (SNFs), e-stim supports elderly patients by improving tolerance to therapy, reducing discomfort, and helping maintain muscle strength [3,4].

This case presentation explores the role of e-stim as an adjunctive therapy for older adults recovering from shoulder trauma in SNF settings.

## Case presentation

An 87-year-old woman with a past medical history of hypertension, hyperlipidemia, osteoporosis, hearing loss, and memory impairment presented to the emergency department via ambulance after sustaining a fall at home while exiting the shower. During the fall, she attempted to stabilize herself by grabbing a towel rack with her right arm, which subsequently gave way, resulting in a fall onto her right side.

Upon arrival, she reported excruciating pain in the right torso and right upper extremity. Physical examination revealed significant ecchymosis over the right shoulder and lateral chest wall, as well as severely limited range of motion of the right shoulder, with pain elicited on any attempted movement.

**Figure 1:**
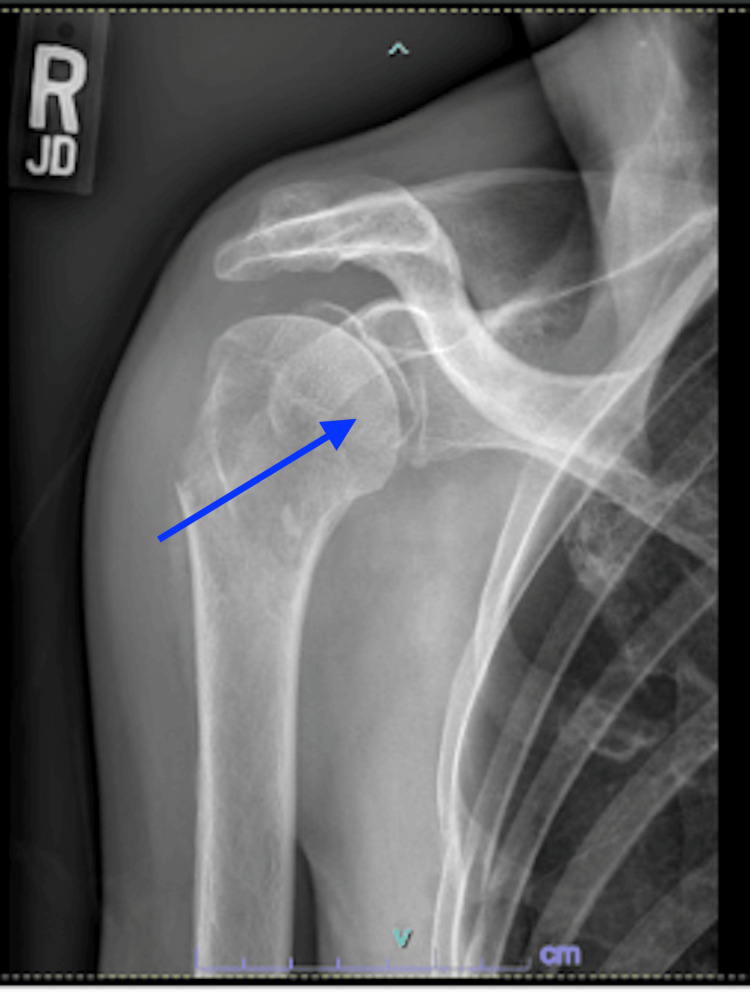
X-ray of the right shoulder on 04/13/2025, showing posterior dislocation of the humeral head and displaced glenoid fracture. This image was taken prior to closed reduction under anesthesia.

**Figure 2:**
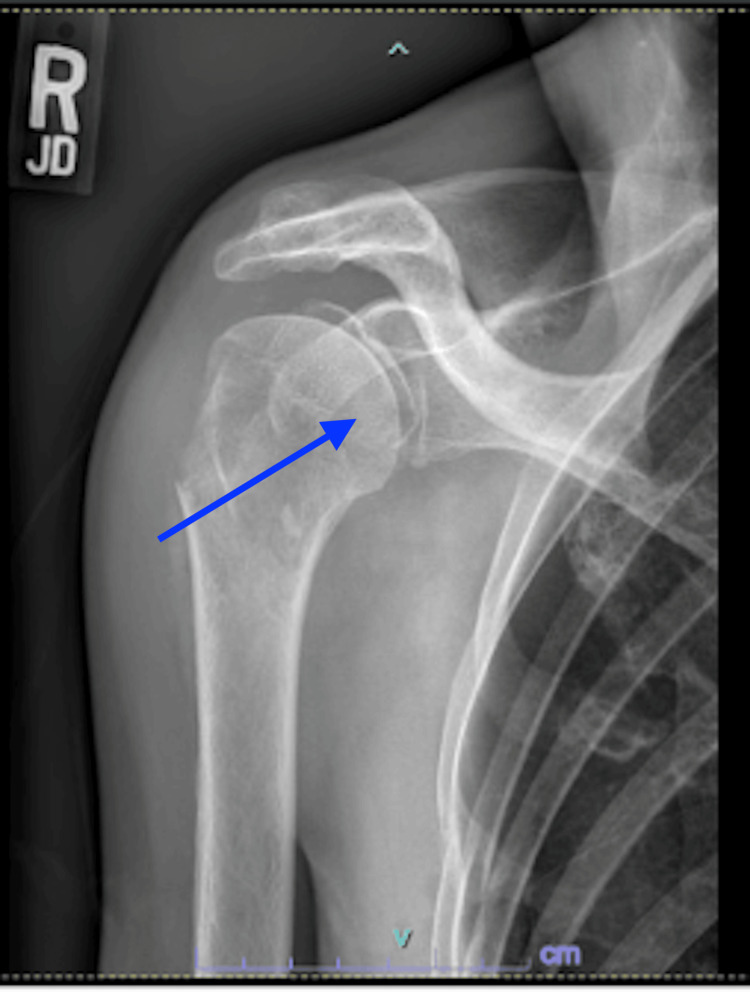
X-ray of the right shoulder on 04/29/2025, demonstrating the concentrically reduced humeral head with medially displaced greater tuberosity fracture, post reduction.

Emergency department workup was notable for multiple right-sided rib fractures on imaging combined with a posterior dislocation of the right humeral head, right greater tuberosity fracture, and an 11 mm right glenoid fracture after a mechanical fall. The patient received right shoulder closed reduction under anesthesia at the hospital on April 13, 2025 (Figures 1-2).

After hospital discharge, she went to a skilled nursing facility for ongoing rehabilitation. Imaging tests at follow-up showed that the greater tuberosity fracture was medially displaced, while the humeral head was concentrically reduced. Orthopedic surgery decided not to perform surgery because of her age and medical conditions and the good alignment of the shoulder and recommended a conservative approach with physical therapy, activity restriction, and follow-up imaging.

During her stay at the SNF, she remained non-weight bearing on her right arm per Orthopedics recommendations and received physical and occupational therapy. Physical and occupational therapy were provided in hourly sessions, five days per week at the SNF, and included passive and active-assisted range of motion exercises for the elbow, wrist, and hand, along with resistance band strengthening and early joint mobilization techniques. Due to significant pain limiting functional progress, electrical stimulation (e-stim) therapy was incorporated into her treatment plan under the direction of the therapy team to enhance pain control, local circulation, and neuromuscular re-education.

**Figure 3:**
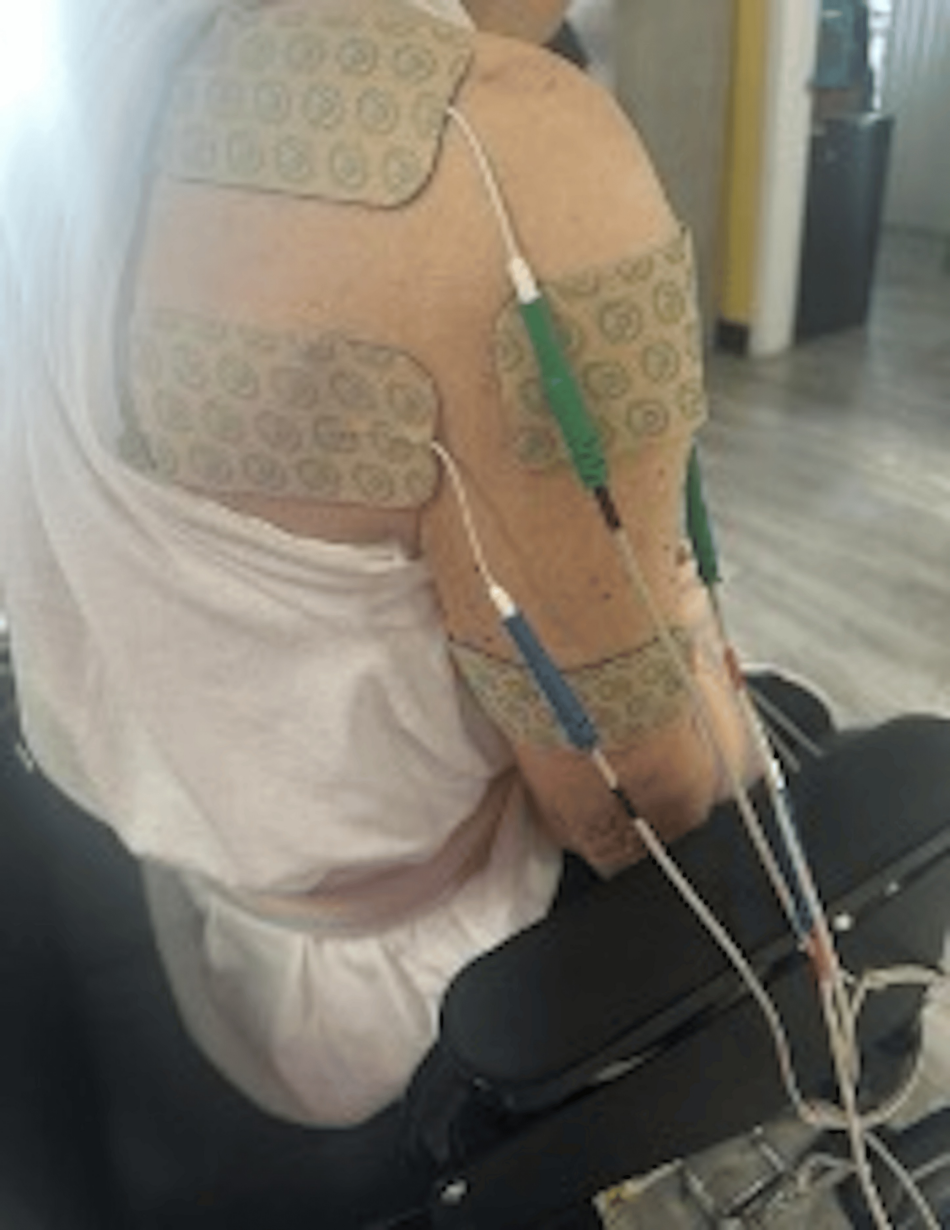
E-stim therapy session targeting the periscapular region, performed while the patient was seated in her wheelchair.

**Figure 4:**
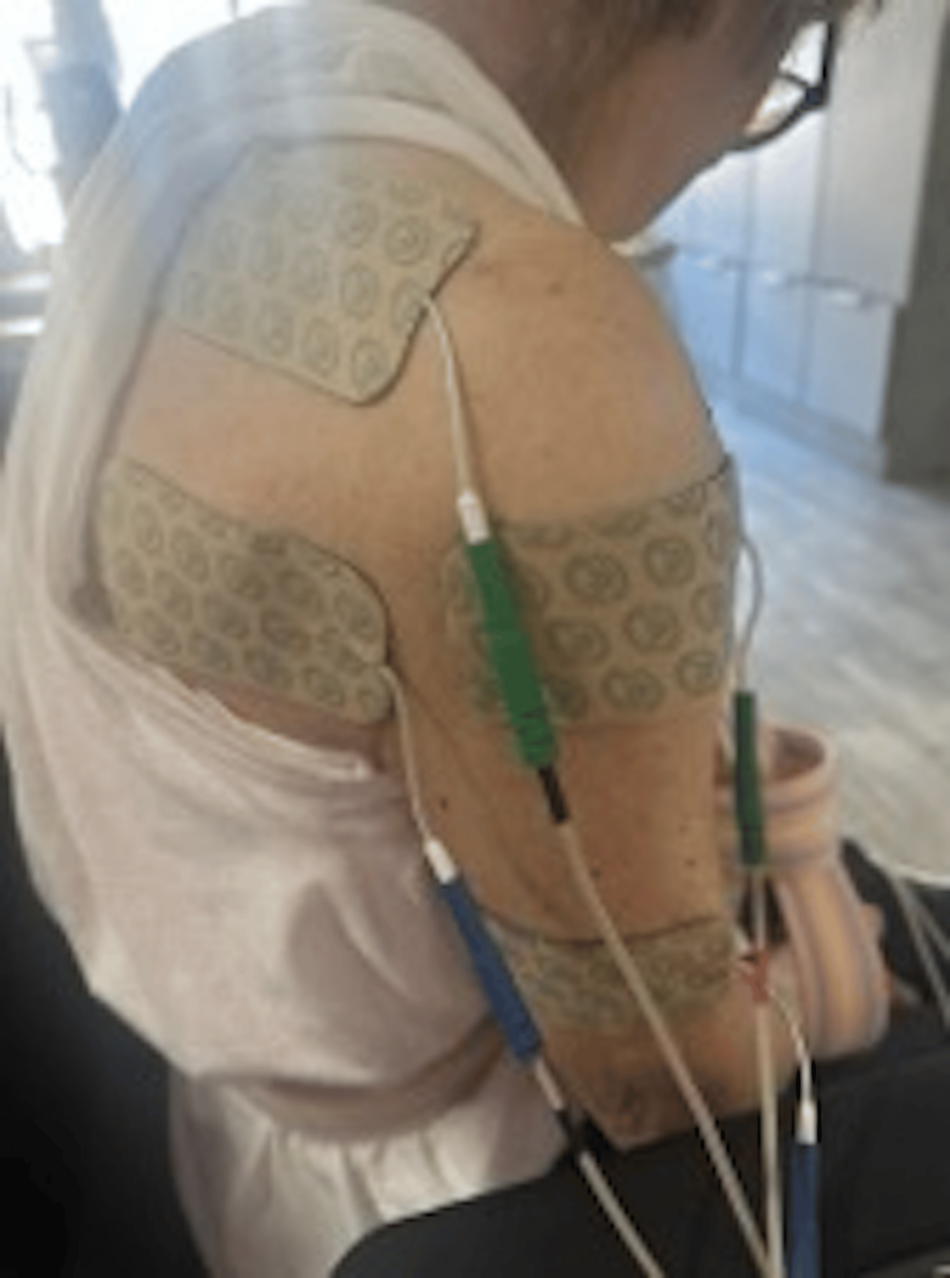
Application of e-stim pads over the right deltoid during therapy session. Electrodes were placed to facilitate neuromuscular re-education and pain control.

The sessions were conducted under the supervision of a therapist and focused on the trapezius, rhomboids, rotator cuff, and deltoid muscles (Figures 3-4). She experienced no pain during treatment, and she was able to participate better in therapy and daily activities. She moved from being unable to move to being able to sit upright for longer periods and to use her left upper extremity for daily activities while keeping the right side protected.

## Discussion

Recovery from shoulder dislocations and glenoid fractures in elderly patients is often prolonged, with a heightened risk of lasting functional impairment [1]. In medically complex individuals, conservative management is frequently favored; however, outcomes can be adversely impacted by persistent pain, immobility, and sarcopenia. Thus, early mobilization and effective pain control are critical for preserving function and preventing deconditioning [2].

E-stim is a widely utilized modality in rehabilitation medicine, aimed at reducing pain and enhancing muscle activation. Neuromuscular electrical stimulation (NMES) has been shown to mitigate muscle atrophy and preserve muscle mass during periods of immobilization [3]. Transcutaneous electrical nerve stimulation (TENS), a subtype of e-stim, has demonstrated efficacy in managing musculoskeletal pain among older adults [4]. A recent meta-analysis further supports the use of NMES, indicating significant improvements in functional recovery and range of motion when integrated into physical therapy regimens for elderly populations [5]. Incorporating NMES into rehabilitation protocols can improve patient outcomes by enhancing muscle strength and motor control, potentially shortening lengths of stay for individuals admitted for similar injuries in skilled nursing facilities (SNFs). In post-operative orthopedic care, e-stim has also been associated with enhanced patient engagement, improved comfort, and superior functional outcomes - particularly when employed as an adjunct to conventional therapy [6].

This case underscores the importance of integrating e-stim into routine rehabilitation protocols at SNFs as a non-invasive and well-tolerated adjunct therapy that can enhance recovery in elderly patients. This population frequently encounters unique challenges to rehabilitation, including frailty, polypharmacy, and pain - factors that e-stim may effectively help mitigate. By addressing these barriers, e-stim has the potential to improve functional outcomes and reduce lengths of stay for patients recovering from similar injuries.

## Conclusions

The use of e-stim therapy in this SNF patient with a right glenoid fracture and humeral head dislocation appeared to facilitate a faster rehabilitation process and improved pain management. This case highlights the potential of e-stim as a non-invasive, well-tolerated adjunct to physical therapy for elderly patients with complex shoulder injuries, particularly in settings where early mobilization and effective pain control are critical to long-term recovery.
